# Supercoiled fibres of self-sorted donor–acceptor stacks: a turn-off/turn-on platform for sensing volatile aromatic compounds†

**DOI:** 10.1039/c6sc00629a

**Published:** 2016-03-21

**Authors:** Anjamkudy Sandeep, Vakayil K. Praveen, Kalathil K. Kartha, Venugopal Karunakaran, Ayyappanpillai Ajayaghosh

**Affiliations:** a Photosciences and Photonics Section, Chemical Sciences and Technology Division, CSIR-National Institute for Interdisciplinary Science and Technology (CSIR-NIIST) Thiruvananthapuram 695 019 India ajayaghosh@niist.res.in; b Academy of Scientific and Innovative Research (AcSIR), CSIR-NIIST Campus Thiruvananthapuram 695 019 India

## Abstract

To ensure the comfortable survival of living organisms, detection of different life threatening volatile organic compounds (VOCs) such as biological metabolites and carcinogenic molecules is of prime importance. Herein, we report the use of supercoiled supramolecular polymeric fibres of self-sorted donor–acceptor molecules as “turn-off/turn-on” fluorescent sensors for the detection of carcinogenic VOCs. For this purpose, a *C*_3_-symmetrical donor molecule based on oligo(*p*-phenylenevinylene), *C*_3_OPV, and a perylene bisimide based acceptor molecule, *C*_3_PBI, have been synthesized. When these two molecules were mixed together in toluene, in contrast to the usual charge transfer (CT) stacking, supramolecular fibres of self-sorted stacks were formed at the molecular level, primarily driven by their distinct self-assembly pathways. However, CT interaction at the macroscopic level allows these fibres to bundle together to form supercoiled ropes. An interfacial photoinduced electron transfer (PET) process from the donor to the acceptor fibres leads to an initial fluorescence quenching, which could be modulated by exposure to strong donor or acceptor type VOCs to regenerate the respective fluorescence of the individual molecular stacks. Thus, strong donors could regenerate the green fluorescence of *C*_3_OPV stacks and strong acceptors could reactivate the red fluorescence of *C*_3_PBI stacks. These supercoiled supramolecular ropes of self-sorted donor–acceptor stacks provide a simple tool for the detection of donor- or acceptor-type VOCs of biological relevance, using a “turn-off/turn-on” fluorescence mechanism as demonstrated with *o*-toluidine, which has been reported as a lung cancer marker.

## Abstract 2

Supercoiled fibres of self-sorted donor–acceptor assemblies that allow the sensing of nitroaromatics and aromatic amines using a “turn-off/turn-on” mechanism are reported.

## Graphical abstract

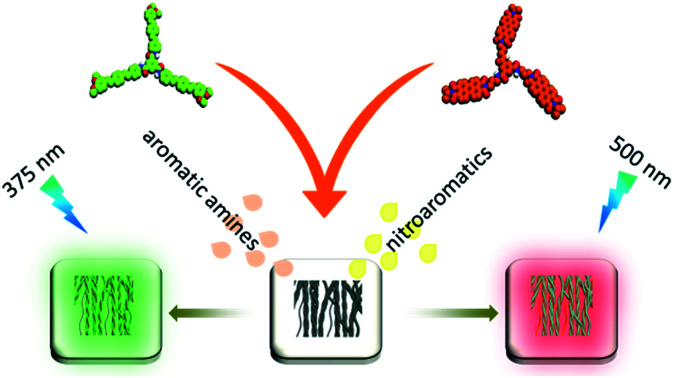

## Introduction

Early detection of deadly diseases such as cancer can save the lives of millions of people across the globe and hence is a prime concern of scientists and clinicians. At the onset of certain diseases, the metabolism of the human body changes to produce several volatile organic compounds (VOCs) in small quantities, some of which can be designated as disease markers.^1^ Detection of cancer markers and carcinogenic VOCs such as *o*-toluidine, aromatic amines, nitroaromatics *etc.* is important since tobacco smoke contains a large number of them, which are known to cause bladder cancer.^2*a*^*o*-Toluidine has also been detected in exhaled air from lung cancer patients.^2*b*^ Similarly, detection of electron deficient molecules such as nitroaromatics is important since they are not only considered as explosives but are also toxic to living organisms through contamination of air and water.^3^

Considering the social relevance of the detection of carcinogenic VOCs, intense research is needed for further development in this area. These considerations prompted us to explore the potential of fluorescent donor–acceptor assemblies designed based on the principles of nanoarchitectonics^4^ for the sensing of volatile analytes. A number of reports are available on the sensing of VOCs such as aromatic amines^5^ and nitroaromatics^3,6,7^ that generally cause fluorescence quenching of a probe. In this context, self-sorted supramolecular assemblies^8–10^ are an ideal platform for the sensing of VOCs. We have earlier shown that fluorescent π-gelators are powerful tools for attogram level sensing of trinitrotoluene (TNT) through a contact mode^7*a*^ and thought that there could be immense scope for expanding this idea to the sensing of VOCs of metabolic origin, if the principles of molecular self-assembly and self-sorting are combined.

**Fig. 1:**
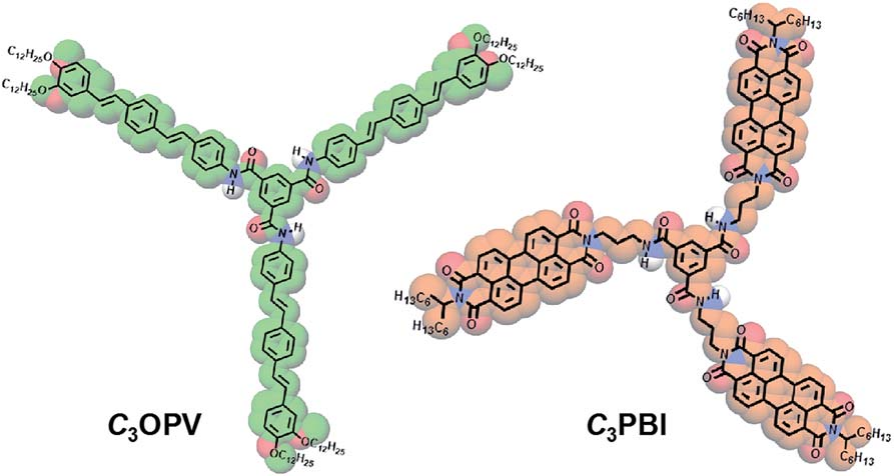
Chemical structures and molecular models (shown in colour) of *C*_3_OPV and *C*_3_PBI.

Usually, when donor and acceptor monomers are mixed, CT induced supramolecular polymers are formed.^11^ Supramolecular control of the polymerization is difficult in such cases.^12^ However, suitably functionalized π-systems^10^ such as oligo(thiophenes) (OTs), oligo(*p*-phenylenevinylenes) (OPVs) and perylene bisimides (PBIs) are known to form self-sorted supramolecular polymeric stacks when mixed, in which the emission is quenched due to photoinduced electron transfer (PET) from the electron rich OTs or OPVs to the electron deficient PBIs.^10*a*,*b*^ Recently, we reported the formation of self-sorted supramolecular assemblies of thienylenevinylenes and PBIs that form coaxial fibres^10*d*^ through weak interfacial charge transfer interactions.^13^ Based on these findings, we hypothesized that suitably designed *C*_3_-symmetrical systems of OPVs and PBIs may form supramolecular polymers of self-sorted donor and acceptor fibres with quenched fluorescence. In such a case, the weak interfacial donor–acceptor interactions in the supercoiled fibres at supramolecular level can be perturbed by exposure to strong donor or acceptor molecular vapours, which may result in a “turn-on” fluorescence with distinct colour variation. As a proof-of-concept for this hypothesis, we illustrate that a combination of a *C*_3_-symmetrical OPV, *C*_3_OPV, and a *C*_3_-symmetrical PBI, *C*_3_PBI, (Fig. 1) forms supercoiled fibres of self-sorted donor–acceptor stacks, which results in a “turn-off/turn-on” fluorescence sensor for the detection of different aromatic VOCs.

## Results and discussion

**Fig. 2:**
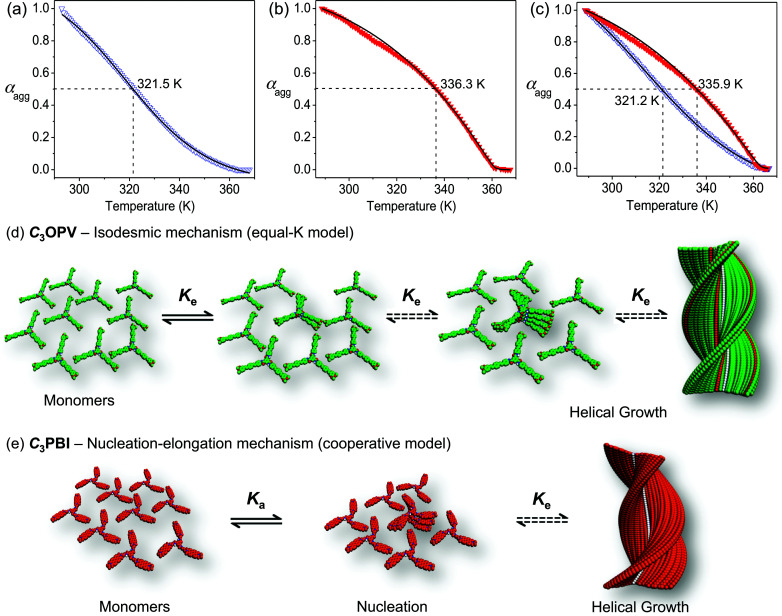
Plot of the fraction of aggregates (*α*_agg_) against temperature for (a) *C*_3_OPV and (b) *C*_3_PBI individual assemblies, and (c) for *C*_3_OPV and *C*_3_PBI as a 1 : 1 mixture in toluene (1 × 10^−4^ M). *C*_3_OPV (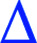), *C*_3_PBI (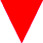) and curve fitting (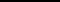). Absorbance was monitored at 425 and 527 nm for *C*_3_OPV and *C*_3_PBI, respectively, with a rate of cooling of 1 K min^−1^. Schematic illustration of the self-assembly pathway of (d) *C*_3_OPV and (e) *C*_3_PBI. *K*_e_ is the association constant and *K*_a_ is the activation constant expressing the degree of cooperativity.

Synthesis of *C*_3_OPV and *C*_3_PBI was accomplished as shown in Schemes S1 and S2,† respectively, and they were characterised using FT-IR, ^1^H and ^13^C NMR spectroscopy, and MALDI-TOF mass spectrometry. Having obtained these molecules in a pure form, our first objective was to get a clear idea of the mechanistic pathway for the individual assembly of *C*_3_OPV and *C*_3_PBI. Detailed UV/Vis absorption studies revealed that these molecules self-assemble in toluene at a concentration range of 10^−4^ to 10^−5^ M (Fig. S1†). Further understanding of the self-assembly mechanism was possible from temperature-dependent absorption studies. For this purpose, the change in the absorption shoulder band at 425 nm of a hot toluene solution of (1 × 10^−4^ M) *C*_3_OPV was monitored as a function of temperature with a cooling rate of 1 K min^−1^ (Fig. S1b†). No hysteresis was observed when the solution was heated again to the monomeric state, indicating that the self-assembly process is reversible. It was clear from the plot of the fraction of aggregates (*α*_agg_) *versus* temperature that the molecule forms assemblies through an isodesmic pathway (equal-K model) as indicated by the broad melting curve, which could be fitted to a standard isodesmic model (Fig. 2a).^14^ This observation is quite surprising, especially considering the fact that most of the *C*_3_-symmetrical benzene trisamide derivatives are known to self-assemble through a cooperative nucleation–elongation mechanism.^15^ Based on this observation, we concluded that the isodesmic self-assembly of *C*_3_OPV (Fig. 2d) presumably is governed by π–π stacking of the OPV moieties and that the contribution from directional intermolecular H-bonding may be weak due to the presence of a sterically demanding aromatic core and six alkyl chains at the periphery.^15,16^ The thermodynamic parameters were calculated by applying the isodesmic model and are summarized in Table 1. The melting transition temperature (*T*_m_, temperature at which *α*_agg_ = 0.50) of the assembly was found to be 321.5 K (Fig. 2a) with an enthalpy value of −85.1 kJ mol^−1^ and an association constant of 4.7 × 10^4^ M^−1^.

**Table 1:** Thermodynamic parameters for the self-assembly of *C*_3_OPV obtained using the isodesmic model^a^

*C*_3_OPV	*C* (mM)	Δ*H* (kJ mol^−1^)	Δ*S* (J mol^−1^ K^−1^)	*T*_m_ (K)	*K*_e_ (10^4^ M^−1^)	DP_N_
Alone	0.1	−85.1	−194.5	321.5	4.7	2.7
In the mixture	0.1	−73.6	−158.0	321.2	3.7	2.5

a *C* is the concentration, Δ*H* is the change in enthalpy, Δ*S* is the change in entropy, *T*_m_ is the melting transition temperature, *K*_e_ is the association constant and DP_N_ is the degree of polymerization.

To probe the self-assembly pathway of *C*_3_PBI, the absorption changes at 527 nm were monitored as a function of temperature with a cooling rate of 1 K min^−1^ (Fig. S1d†). The plot of *α*_agg_ with temperature showed a non-sigmoidal transition, characteristic of a cooperative pathway, which could be fitted to a nucleation–elongation model (Fig. 2b and e).^14*c*,15*a*,17–19^ By applying this model, the elongation temperature (*T*_e_) was determined as 360.5 K and the enthalpy release upon elongation (*H*_e_) was calculated as −27.4 kJ mol^−1^. A high degree of cooperativity (*K*_a_) was inferred from the small value of the equilibrium constant (10^−6^) for the nucleation step.

After obtaining an idea of the individual assembly mechanisms of *C*_3_OPV and *C*_3_PBI in toluene, we studied the effect of mixing these molecules at a 1 : 1 ratio by monitoring the changes in the absorption spectra under identical experimental conditions. The resultant spectrum of the mixture in toluene (1 × 10^−4^ M) was found to be a sum of the absorption spectra of the individual constituents (Fig. S2†). Furthermore, the absence of a CT band in the absorption spectrum excludes the possibility of a molecular level donor–acceptor interaction. The mixture was cooled down slowly at a rate of 1 K min^−1^. The variable temperature absorption spectral changes of the mixture monitored at 425 and 527 nm exhibited that melting of the individual aggregates occurred without much variation from their respective melting transition curves as observed for the individual assemblies (Fig. 2c and S3†). The transition curves obtained from a plot of *α*_agg_*versus* temperature could be fitted to an isodesmic model and nucleation–elongation model for *C*_3_OPV and *C*_3_PBI, respectively (Fig. 2c). The thermodynamic parameters calculated for the mixture from the curve fitting are in good agreement with that of the individual assemblies. The melting transition temperature, *T*_m_, of *C*_3_OPV in the mixture is 321.2 K, which is close to that of *C*_3_OPV alone (321.5 K). Similarly, *T*_m_ of the *C*_3_PBI assemblies in the mixture is 335.9 K, which matches to that observed for the individual assembly of *C*_3_PBI (336.3 K) (Fig. 2). The other thermodynamic parameters such as enthalpy and entropy changes of the molecules in the mixture also match with those of the individual molecular assemblies (Tables 1 and 2). These results imply that both *C*_3_OPV and *C*_3_PBI form self-sorted stacks when they are mixed.

**Table 2:** Thermodynamic parameters for the self-assembly of *C*_3_PBI obtained using the nucleation elongation model^a^

*C*_3_PBI	*C* (mM)	Δ*H*_e_ (kJ mol^−1^)	Δ*S*_e_ (J mol^−1^ K^−1^)	*T*_m_ (K)	*T*_e_ (K)	*K*_a_
Alone	0.1	−27.4	−140.9	336.3	360.5	10^−6^
In the mixture	0.1	−27.5	−132.6	335.9	362.4	10^−6^

a *C* is the concentration, Δ*H*_e_ and Δ*S*_e_, respectively, are the change in enthalpy and entropy during the elongation process, *T*_m_ is the melting transition temperature, *T*_e_ is the elongation temperature and *K*_a_ is the degree of cooperativity.

Other important parameters for supporting the formation of a self-sorted assembly are the association constant (*K*_e_) in the case of *C*_3_OPV and the degree of cooperativity (*K*_a_) in the case of *C*_3_PBI (Tables 1 and 2). The association constant for the addition of individual monomers to the growing assembly of *C*_3_OPV in the mixture is 3.7 × 10^4^ M^−1^, which almost matches with the value for the individual assembly of *C*_3_OPV (4.7 × 10^4^ M^−1^). For the cooperative self-assembly of *C*_3_PBI, the degree of cooperativity is in the range of 10^−6^, similar to that of the individual assembly.

**Fig. 3:**
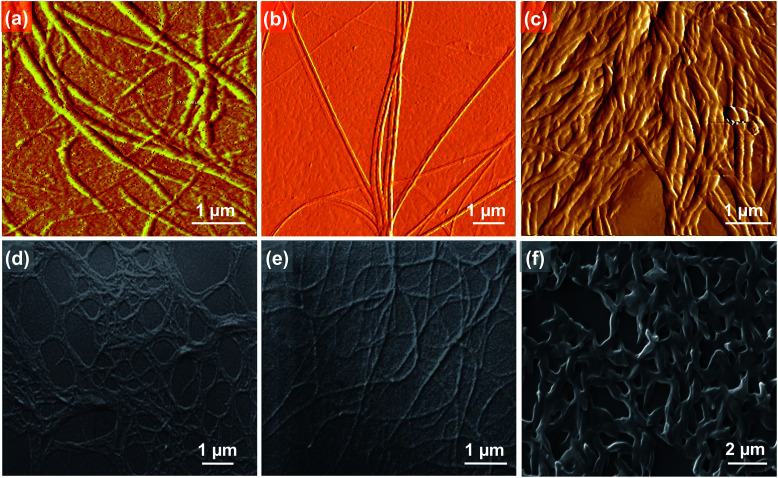
AFM (a), (b) and (c), and SEM (d), (e) and (f) images of *C*_3_OPV, *C*_3_PBI, and the 1 : 1 mixture, respectively.

**Fig. 4:**
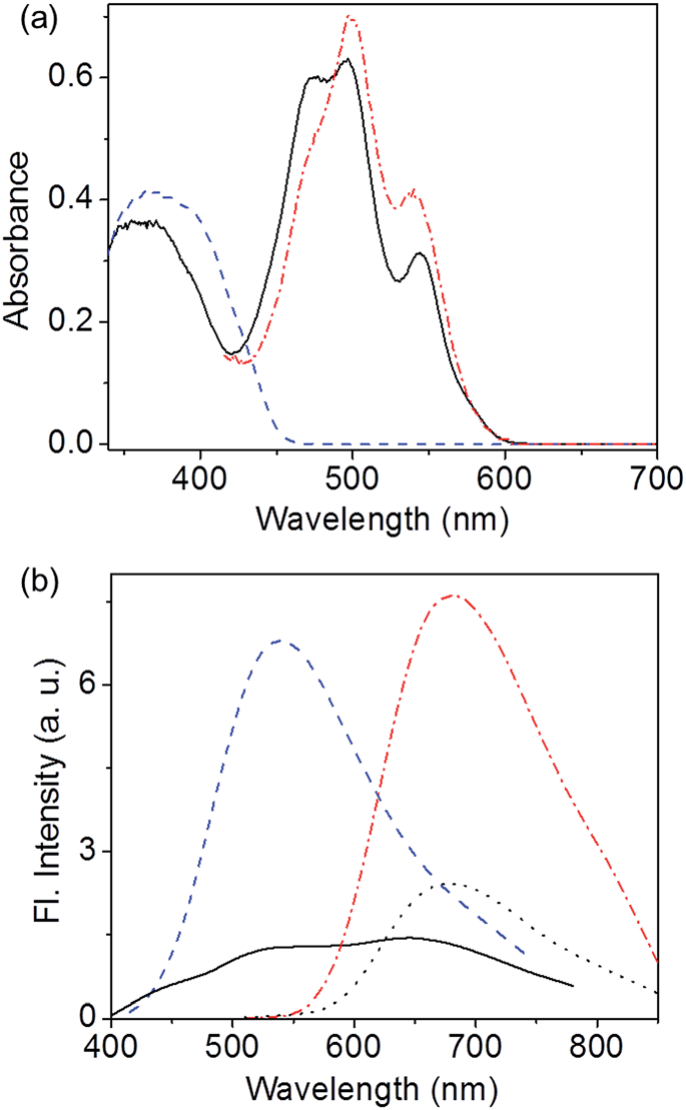
(a) Absorption spectra of *C*_3_OPV (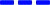), *C*_3_PBI (
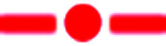
), and a 1 : 1 mixture of *C*_3_OPV and *C*_3_PBI (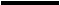) in the film state. (b) Emission spectra of *C*_3_OPV (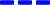), *λ*_ex_ = 375 nm, *C*_3_PBI (
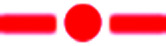
), *λ*_ex_ = 500 nm, and a 1 : 1 mixture of *C*_3_OPV and *C*_3_PBI (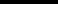), *λ*_ex_ = 375 nm and (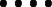) *λ*_ex_ = 500 nm, in the film state.

Atomic force microscopy (AFM) images of *C*_3_OPV drop cast from a 1 × 10^−4^ M toluene solution on a freshly cleaved mica surface revealed the formation of micrometer long helical fibres of a diameter of 200–250 nm (Fig. 3a). *C*_3_PBI also displayed the formation of helical fibres with the diameter varying from 100–150 nm and the length extended to several micrometres (Fig. 3b). For the 1 : 1 mixture assembly, the fibre-like morphology was retained, however, the formation of supercoiled helical ropes with an increased diameter (400–500 nm) was observed (Fig. 3c). The scanning electron microscopy (SEM) images also support the formation of helical fibres and supercoiled ropes (Fig. 3d–f). These observations are in analogy with previous reports on the self-assembly of *C*_3_-symmetrical *N*,*N*′,*N*′′-trialkyl benzene-1,3,5-tricarboxamide in which the amide functionality is involved in a three-fold helical array of intermolecular hydrogen bonding.^20^ From the mechanistic studies and the morphological features, it was inferred that self-sorted donor and acceptor fibres are formed initially, which enter into weak interfacial charge transfer interactions at the supramolecular level resulting in supercoiled ropes.^10*d*,13^ The absorption spectra of a 1 : 1 mixture of *C*_3_OPV and *C*_3_PBI in solution (toluene, 1 × 10^−4^ M, Fig. S2†) and film states (Fig. 4a) did not show any CT bands, indicating the absence of a molecular level donor–acceptor interaction. However, significant quenching of the individual emissions of *C*_3_OPV and *C*_3_PBI was observed in the solution and the film states (Fig. S4† and 4b). These observations could be ascribed to a possible PET from the donor OPV to the acceptor PBI.

**Fig. 5:**
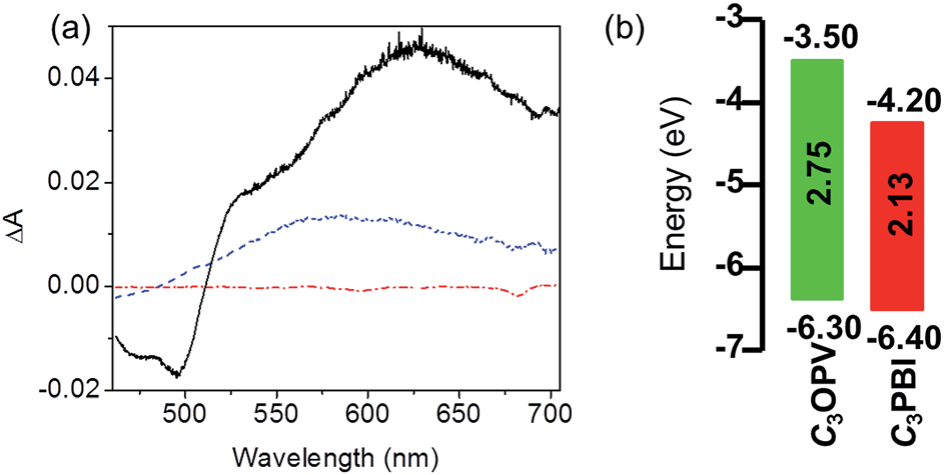
(a) Transient absorption spectra of *C*_3_OPV (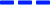), *C*_3_PBI (
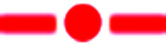
) and a 1 : 1 mixture (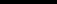) in toluene (1 × 10^−4^ M) recorded at 1.9 ps, *λ*_ex_ = 380 nm. (b) An energy level diagram for *C*_3_OPV and *C*_3_PBI.

The PET process between *C*_3_OPV and *C*_3_PBI was investigated using femtosecond pump-probe spectroscopy. When a solution containing a 1 : 1 mixture of *C*_3_OPV and *C*_3_PBI was excited at 380 nm, where mainly *C*_3_OPV absorbs, the transient absorption spectra showed the formation of a radical anion of *C*_3_PBI absorbing broadly around 630 nm with a decay time of around 728 ps (Fig. 5a), which indicated PET from the OPV to the PBI.^21^ The feasibility of PET between these molecules was further established using photoelectron yield spectroscopic studies (Fig. S5†). From the value for the HOMO and the optical band gap (*E*_g_) obtained from the film state absorption spectrum (Fig. S6†), the LUMO values of both *C*_3_OPV and *C*_3_PBI were calculated. *C*_3_PBI showed a slightly lower LUMO (−4.20 eV) when compared to that of *C*_3_OPV (−3.50 eV) (Fig. 5b). Since *C*_3_PBI is an electron accepting molecule compared to *C*_3_OPV, the HOMO level of the former is lower than that of *C*_3_OPV (Fig. 5b). Therefore, upon photoexcitation, electrons are transferred from *C*_3_OPV to *C*_3_PBI leading to quenching of the emission.

**Fig. 6:**
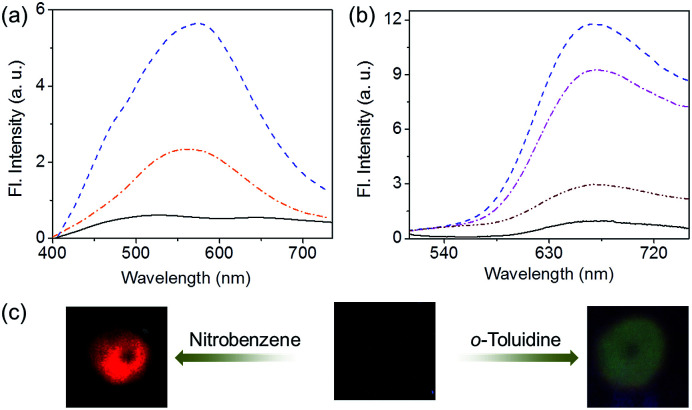
The emission spectra of films of a 1 : 1 mixture of *C*_3_OPV and *C*_3_PBI before and after exposure to vapours of different (a) aromatic amines [(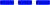) *o*-toluidine, (
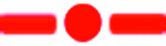
) aniline and (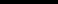) blank)], *λ*_ex_ = 375 nm, and (b) nitroaromatics [(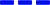) nitrobenzene, (
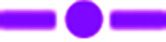
) 2-nitrotoluene, (
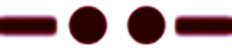
) 2,4-dinitrotoluene and (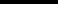) blank], *λ*_ex_ = 500 nm. (c) Photographs showing the fluorescence of the films of a 1 : 1 mixture of *C*_3_OPV and *C*_3_PBI before and after exposure to different volatile aromatic compounds.

**Fig. 7:**
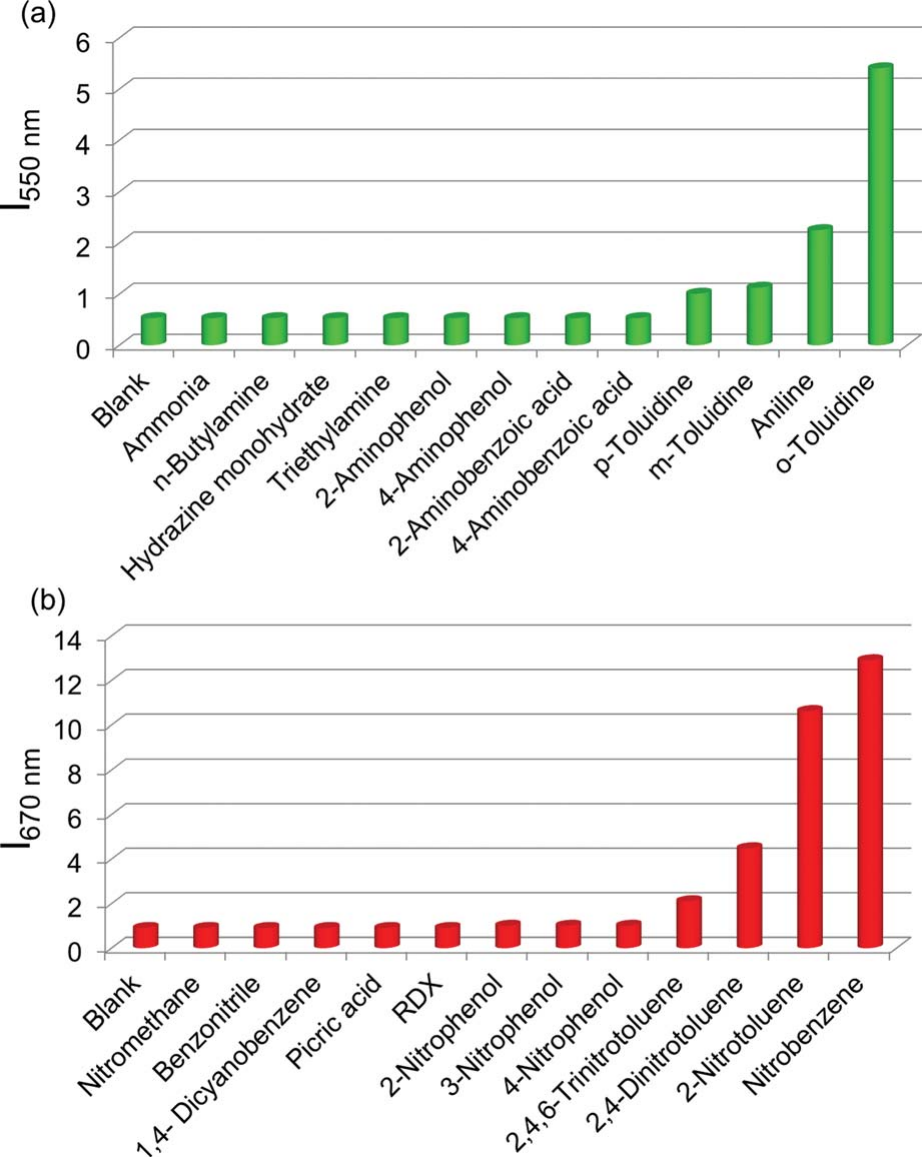
Selectivity plots for the vapour phase detection of different volatile (a) amines and (b) nitro compounds using films prepared from a 1 : 1 mixture of *C*_3_OPV and *C*_3_PBI. The emission of *C*_3_OPV was monitored at 550 nm (*λ*_ex_ = 375 nm) for the aromatic amine exposed films and that of *C*_3_PBI was monitored at 670 nm (*λ*_ex_ = 500 nm) in the case of the nitro compound exposed films. In all these studies, the films were exposed to VOCs for 2 min.

Since OPVs are known to interact with electron deficient aromatic nitro compounds^3*e*,7*a*^ and PBIs with electron rich aromatic amines,^5*a*–*c*,22^ we thought that the quenched emission of the supercoiled *C*_3_OPV and *C*_3_PBI fibres could be “turned on” when they come into contact with a better donor or an acceptor molecule. In order to prove this hypothesis, a toluene solution of a 1 : 1 mixture of *C*_3_OPV and *C*_3_PBI (20 μL of a 10^−3^ M solution) was drop cast on glass substrates and exposed to various analytes. The film that was exposed to aromatic amines such as *o*-toluidine displayed a greenish-yellow emission (Fig. 6a and c). Comparison of the absorption spectrum of *C*_3_OPV and the excitation spectrum obtained upon monitoring the emission at 540 nm for the 1 : 1 mixture of *C*_3_OPV and *C*_3_PBI revealed that the emission originates from *C*_3_OPV molecules in the mixture (Fig. S7†). On the other hand, a red emission was obtained when the film was exposed to nitrobenzene vapours (Fig. 6b and c). An excitation spectrum of the 1 : 1 mixture monitored at 650 nm showed a resemblance to the absorption spectrum of *C*_3_PBI individual assembly (Fig. S8†), which proves that the red emission is from the self-assembled *C*_3_PBI molecules. Similar experiments were conducted for other aromatic amines such as 2-aminophenol, aniline, *m*-toluidine, *etc.* and nitroaromatics such as TNT, dinitrotoluene (DNT), *o*-nitrotoluene, *etc.*, and the results are summarized in Fig. 7.

**Fig. 8:**
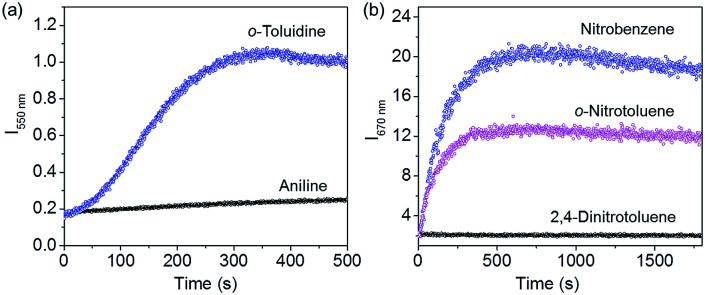
Plots of the emission intensity (a) at 550 nm (*λ*_ex_ = 375 nm) and (b) at 670 nm (*λ*_ex_ = 500 nm) as a function of time after exposing a film prepared from a 1 : 1 mixture of *C*_3_OPV and *C*_3_PBI to the vapour of aromatic amines and nitroaromatics, respectively.

The observed “turn-on” emission for the 1 : 1 mixed *C*_3_OPV and *C*_3_PBI films in the presence of the analytes is explained as follows. Electron rich aromatic amines facilitate a strong CT interaction with the electron deficient *C*_3_PBI fibres, which in turn prevents the weak interfacial PET from the *C*_3_OPV fibres to the *C*_3_PBI fibres, thus activating the *C*_3_OPV emission upon excitation at 375 nm. The emission intensity revival monitored at 550 nm with time was found to depend upon the electron donating ability of the amines used (Fig. 8a). For the first 120 seconds of exposure, around a 5-fold increase in the emission intensity for *o*-toluidine was observed, while only a 2-fold increase was noticed for aniline. The inductive effect of the electron donating methyl group in *o*-toluidine makes it a better donor than aniline. The inductive effect decreases in the case of *m*-toluidine as the methyl group is far from the amino group. Not only the electron donating ability of the different amines but also the vapour pressure of the different amines play an important role in the selective detection of *o*-toluidine. The vapour pressure of *o*-toluidine at 25 °C is around 200 Pa and that of aniline and *m*-toluidine is around 89 and 17 Pa, respectively. This high value of the vapour pressure for *o*-toluidine and the electron donating positive inductive effect of the methyl group result in a fast response upon interaction with a film of the mixed assembly.

When vapours of nitroaromatic compounds such as nitrobenzene and nitrotoluene are exposed to the supercoiled fibres of *C*_3_OPV and *C*_3_PBI, a red emission was observed. Electron deficient nitroaromatics can have a strong CT interaction with the electron rich *C*_3_OPV fibres thereby inhibiting the weak interfacial PET from the *C*_3_OPV fibres to the *C*_3_PBI fibres upon excitation of the later at 500 nm. Hence the interaction between the *C*_3_OPV and *C*_3_PBI stacks becomes weaker, thereby the *C*_3_PBI emission is activated by a favouring of the more energetically feasible PET from *C*_3_OPV to the electron accepting nitroaromatic compounds. In this case also, the sensitivity depends upon both the electron accepting ability and the vapour pressure of the nitro compounds. This is evident from a plot of the emission intensity monitored at 670 nm with the time of exposure (Fig. 8b). It was observed that for the first minute of the exposure, nitrobenzene and 2-nitrotoluene showed an almost equal amount of emission recovery. However, upon extended exposure, nitrobenzene provided more emission revival than the nitrotoluene because of its high vapour pressure (20 Pa) and electron accepting ability. Compared to nitrobenzene, the presence of an electron donating methyl group reduces the electron accepting ability of *o*-nitrotoluene. At the same time, molecules such as DNT and TNT, which are more electron deficient than nitrobenzene, showed less response with the film. This observation is explained on the basis of the difference in the vapour pressure of these nitroaromatics. The vapour pressure of TNT and DNT is 0.0165 and 0.0079 Pa, respectively, which is much less than the vapour pressures of nitrobenzene (20 Pa) and 2-nitrotoluene (38 Pa).

**Fig. 9:**
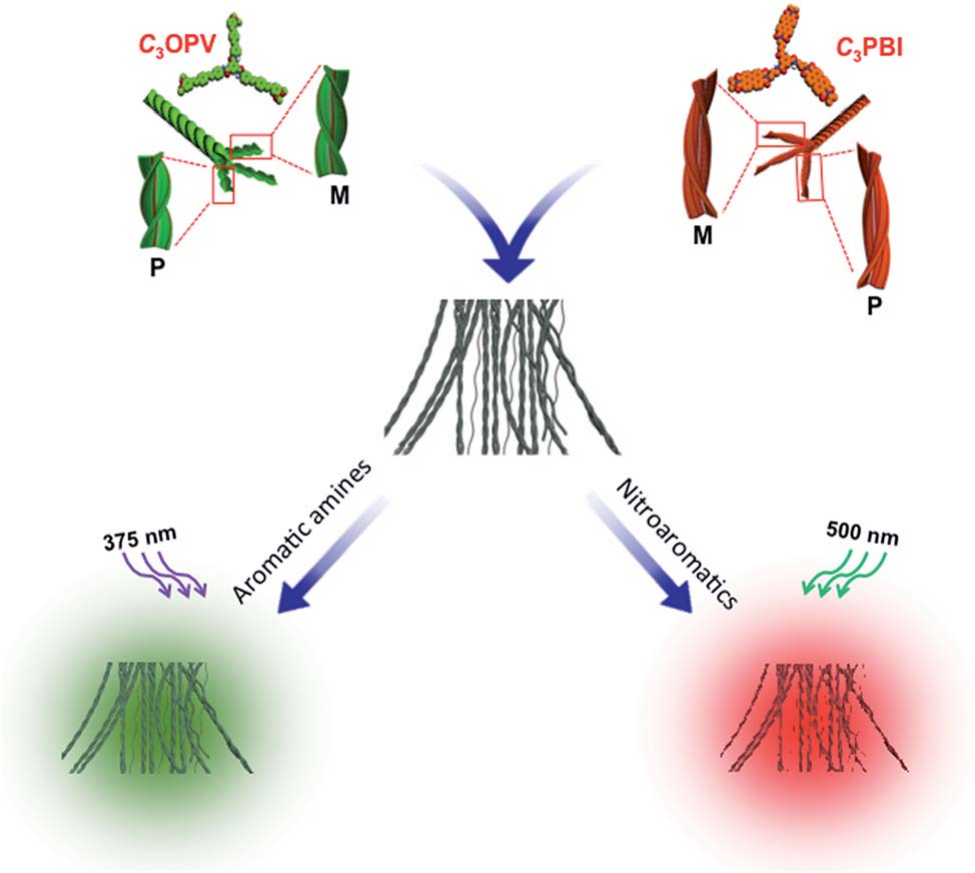
Schematic illustration of the fluorescence ‘turn-off/turn-on’ mechanism of the self-sorted fibres of a 1 : 1 mixture of *C*_3_OPV and *C*_3_PBI on exposure to different VOCs.

The overall process for the sensing of VOCs by supercoiled self-stacks of *C*_3_OPV and *C*_3_PBI is schematically shown in Fig. 9. The *C*_3_ symmetrical OPV and PBI prefer to form columnar helical assemblies of self-sorted stacks. The *C*_3_OPV stacks (green) and the *C*_3_PBI stacks (red), due to weak interfacial CT interactions, bundle together to form supercoiled fibres (black) in which the fluorescence is quenched by PET between the donor–acceptor self-sorted fibres. The PET process is subsequently perturbed by exposing the fibres to strong donor or acceptor molecules, resulting in respective fluorescence signals from *C*_3_OPV or *C*_3_PBI.

## Conclusions

By taking advantage of self-sorting at the molecular level and electronic interactions at the macroscopic level, we could design nonfluorescent supercoiled fibres of *C*_3_OPV and *C*_3_PBI molecules. The self-sorting is facilitated by differences in the self-assembly pathways of the individual molecules wherein *C*_3_OPV followed an isodesmic model and *C*_3_PBI preferred a cooperative mechanism. Interfacial PET between the self-sorted fibres resulted in the quenching of the initial fluorescence of the molecules, which could be perturbed by exposure to VOCs, especially electron rich compounds such as aromatic amines and electron deficient compounds such as nitroaromatics. Thus, the green emission of *C*_3_OPV appeared when the film was exposed to *o*-toluidine and the red emission of *C*_3_PBI was obtained by exposing the film to nitroaromatic vapours. The extent of the emission revival depends on the electron donating ability of the aromatic amines and the electron withdrawing ability of the nitroaromatics, in addition to the vapour pressure of the molecules. The fluorescence “turn-off/turn-on” features of the supercoiled supramolecular fibres of the self-sorted donor–acceptor system described here provide the ability to detect *o*-toluidine of metabolic origin, which is a known lung cancer marker.

## Supplementary Material

SC-007-C6SC00629A-s001
