# Supplementary material for: Supercoiled fibres of self-sorted donor–acceptor stacks: a turn-off/turn-on platform for sensing volatile aromatic compounds
Source: Chem Sci. 2016 Mar 21;7(7):4460–7. doi: 10.1039/c6sc00629a (PMC6014296; doi:10.1039/c6sc00629a)
Supplement: SC-007-C6SC00629A-s001 [file SC-007-C6SC00629A-s001.pdf]

Electronic Supplementary Material (ESI) for Chemical Science.
This journal is © The Royal Society of Chemistry 2016

Supporting Information for

Supercoiled fibres of self-sorted donor-acceptor stacks: a turn-off/turn-on platform for sensing volatile aromatic compounds

Anjamkudy Sandeep,[a] Vakayil K. Praveen,[a] Kalathil K. Kartha,[a] Venugopal Karunakaran[a,b] and Ayyappanpillai Ajayaghosh*[a,b]

[a]Photosciences and Photonics Group, Chemical Sciences and Technology Division, CSIR-National Institute of Interdisciplinary Science and Technology (CSIR-NIIST), Thiruvananthapuram 695019, India.

Email: ajayaghosh@niist.res.in

[b]Academy of Scientific and Innovative Research (AcSIR), CSIR-NIIST Campus, Thiruvananthapuram 695 019, India.

Index

1. Materials and Methods 3
   1.1. Synthesis-General Procedures 3
   1.2. Synthesis-Characterization Techniques 3
   1.3. Measurements 3
   1.4. Scheme for The Synthesis 4
      1.4.1. Synthesis of ***C*$_3$OPV** 4
      1.4.2. Synthesis of ***C*$_3$PBI** 8
   1.5. Isodesmic or Equal-K Self-Association Model 10
   1.6. Nucleation-Elongation Model 11
2. Additional Figures 12
   Figure S1 12
   Figure S2 and S3 13
   Figure S4 and S5 14
   Figure S6 and S7 15
   Figure S8 16
3. Supporting Information References 16

1. Materials and Methods

1.1. Synthesis-General Procedures. Unless otherwise stated, all starting materials and reagents were purchased from commercial suppliers and used without further purification. The solvents were purified and dried by standard methods prior to use. The reactions were monitored using thin layer chromatography (TLC) on silica gel 60 F_{254} (0.2 mm; Merck). Visualization was accomplished using UV lamp (365 nm). Column chromatography was performed on glass columns of different sizes hand packed with silica gel 60 (particle size 0.040–0.063 mm, Merck). Molecules ***C*₃OPV** and ***C*₃PBI** were synthesized according to Scheme S1 and S2 based on standard protocols.

1.2. Synthesis-Characterization Techniques. NMR spectra were measured on a 300 or 500 MHz Bruker Avance DPX spectrometer. Chemical shifts are reported in parts per million (ppm) using tetramethylsilane (TMS) (δ_H = 0 ppm) as an internal reference. The resonance multiplicity is described as s (singlet), d (doublet), t (triplet) and m (multiplet). FT-IR spectra were recorded on a Shimadzu IRPrestige-21 Fourier Transform Infrared Spectrophotometer using KBr pellet method. Mass spectra (MS) were recorded on a JEOLJSM 600 fast atom bombardment (FAB) high-resolution mass spectrometer. Matrix-assisted laser desorption ionization time-of-flight (MALDI-TOF) mass spectra were obtained on a Shimadzu AXIMA-CFR PLUS spectrometer using α-cyano-4-hydroxycinnamic acid as the matrix.

1.3. Measurements. The electronic absorption spectra were recorded on a Shimadzu UV-3101 or 2401PC UV-Vis-NIR scanning spectrophotometer. The fluorescence spectra were recorded on a SPEX-Fluorolog F112X spectrofluorimeter. AFM images were recorded under ambient conditions using a NTEGRA (NT-MDT) operating with a tapping mode regime. Micro-fabricated TiN cantilever tips (NSG10) with a resonance frequency of 299 kHz and a spring constant of S8 20-80 Nm^{-1} was used. Samples for the imaging were prepared by drop casting the sample (1×10^{-4} M) prepared in toluene on freshly cleaved mica surface after drying in vacuum. SEM images were taken on a Zeiss EVO 18 cryo SEM Special Edn with variable pressure detector working at 20–30 kV after sputtering with gold. Samples were prepared by drop casting the aggregates of C_3OPV, C_3PBI and 1:1 mixture in toluene on freshly cleaved mica substrate. It was kept for overnight to allow slow evaporation of the solvent and then further dried in a vacuum desiccator for 12 h. In order to carry out the sensing studies in vapor phase the self-assembled solution were drop cast over glass plates and dried under vacuum. The film was

placed in a chamber containing saturated vapor of the analytes. Emission spectra were collected in a front face geometry using a film sample holder. The samples for photoelectron yield spectroscopy (PYS) were prepared on ITO coated glass plates by solution drop cast (chlorobenzene solution). Prior to the measurements, substrates were dried in a vacuum oven for 2 h at 50 °C. The PYS experiments were done on a RIKEN Keiki Co., Ltd., model AC-3 under high vacuum (1 × 10^{-3} M pa). The instrument was calibrated using standard aluminium substrate.

1.4. Scheme for the Synthesis

1.4.1. Synthesis of C_3OPV

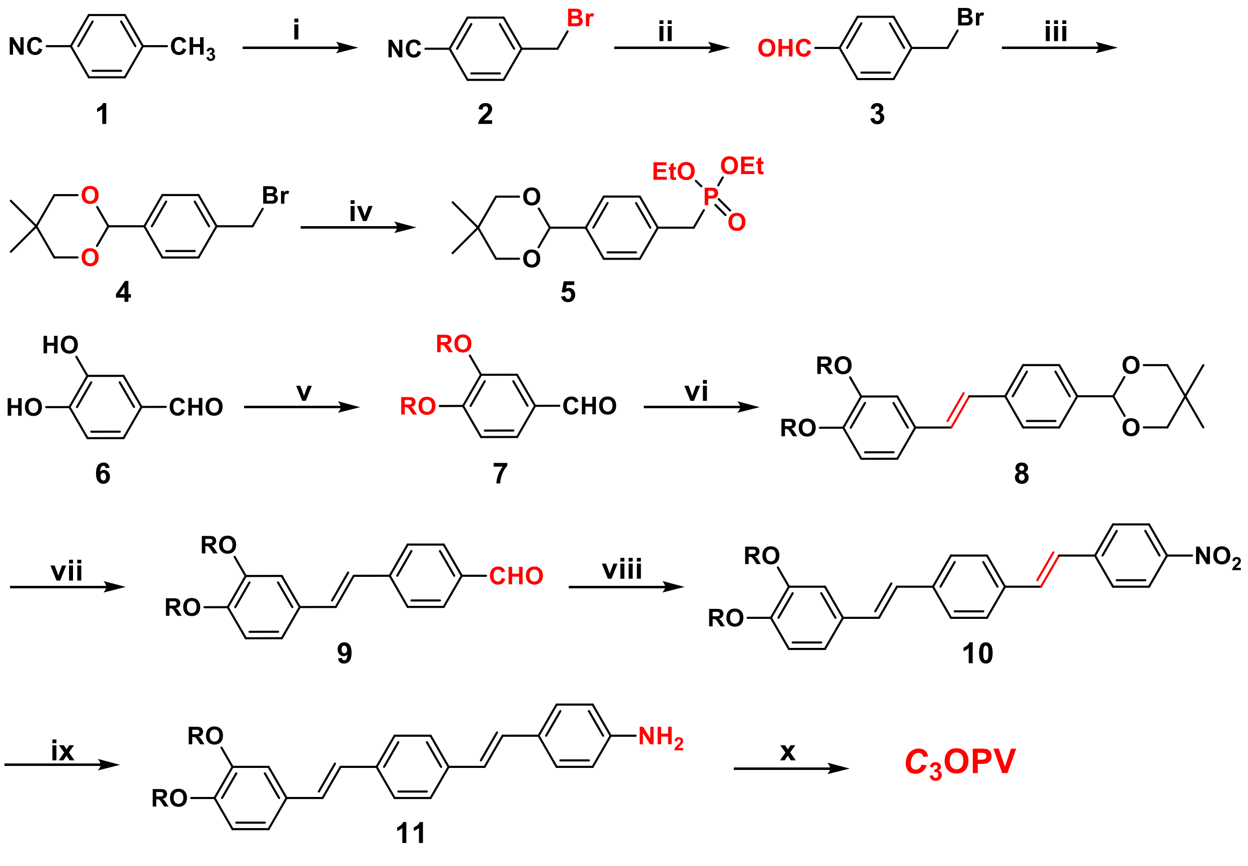

Scheme S1. Reagents and conditions: i) *N*-bromosuccinimide, AIBN, dry CCl_4, 80 ºC, 8 h, 90%; ii) DIBALH, dry toluene, 0 ºC, 1 h, 70%; iii) 2,2-dimethyl-1,3-propanediol, dry benzene, pyridinium hydrochloride (catalytic amount) 100 ºC, 8 h, 80%; iv) triethyl phosphite, 160 ºC, 12 h, 90%; v) 1-bromododecane, K_2CO_3, DMF, 70 ºC, 10 h, 95%; vi) **5**, NaH, dry THF, r.t., 12 h, 85%; vii) TFA, CH_2Cl_2, r.t., 5 h, 90%; viii) diethyl(4-nitrobenzyl)phosphonate, NaH, dry THF, r.t., 2 h, 80%; ix) $SnCl_2 \cdot 2H_2O$, distilled THF, 70 ºC, 4 h, 85%; x) 1,3,5-benzenetricarboxylic acid chloride, Et_3N, dry CH_2Cl_2, r.t., 6 h, 50%.

Preparation of 4-(bromomethyl) benzonitrile (2): To a solution of 4-methylbenzonitrile (**1**) (1 g, 8.32 mmol) in 30 mL of dry CCl_4 were added *N*-bromosuccinimide (1.77 g, 10 mmol) and AIBN. The reaction mixture was refluxed for 8 h. After the completion of the reaction, the hot reaction mixture was cooled and filtered. The solution was then concentrated under reduced pressure, kept overnight for recrystallisation and the product crystallised was filtered and dried. Yield: 90%; ^1H NMR (300 MHz, $CDCl_3$, TMS): δ = 7.63-7.68 (m, 2H), 7.26-7.51 (m, 2H), 4.48 (s, 1H) ppm; MS (FAB): *m/z* calculated for C_8H_6BrN: 196.04; found 196.71.

Preparation of 4-(bromomethyl)benzaldehyde (3): Compound **2** (1 g, 5.1 mmol) was dissolved in 10 mL of dry toluene and cooled at 0 ºC and a portion of 1.08 M DIBALH in hexane (2 equiv.) was added drop wise under nitrogen atmosphere. The solution was stirred for 1 h at 0 ºC. The reaction mixture was first diluted with chloroform (15 mL) followed by 34 mL of 10% HCl and the solution were stirred at room temperature for another 1 h. The organic layer separated was washed with distilled water and dried over anhydrous Na_2SO_4. The solvent was almost completely removed from the filtrate under reduced pressure and the residue was washed with ice-cold *n*-hexane and dried at 50 ºC under vacuum. Yield: 70%; ^1H NMR (300 MHz, $CDCl_3$, TMS): δ = 10.02 (s, 1H), 7.86 (m, 2H), 7.57 (m, 2H), 4.52 (s, 2H) ppm; MS (FAB): *m/z* calculated for C_8H_7BrO: 199.04; found 199.82.

Preparation of 2-(4-bromomethyl)phenyl)-5,5-dimethyl-1,3-dioxane (4): Compound **3** (0.5 g, 1 mmol), 2,2-dimethyl-1,3-propanediol (0.3 g, 2.88 mmol) and catalytic amount of pyridinium hydrochloride were dissolved in dry benzene (30 mL). The solution was then refluxed for 8 h at 100 ºC. The water formed during the reaction was separated using a dean-stark set up. After the completion of the reaction the excess benzene was distilled off and the residue was dissolved in CH_2Cl_2. The solution was then washed with water, dried over anhydrous Na_2SO_4. The product was then further purified using column chromatography over silica gel (30% $CHCl_3$-*n*-hexane). Yield: 80%; ^1H NMR (300 MHz, $CDCl_3$, TMS): δ = 7.61-7.49 (m, 2H), 7.41-7.38 (m, 2H), 5.39 (s, 1H), 4.59 (s, 2H), 3.79-3.63 (dd, 4H), 1.29 (s, 3H), 0.80 (s, 3H) ppm; MS (FAB): *m/z* calculated for $C_{13}H_{17}BrO_2$: 285.18; found 285.90.

Preparation of diethyl 4-(5,5-dimethyl-1,3-dioxan-2-yl)benzylphosphonate (5): A mixture of triethyl phosphite (1.94 g, 11.6 mmol) and **4** (1.25 g, 5.1 mmol) was heated at 160 °C. After 12 h, generated bromoethane and excess triethyl phosphite were distilled off and the residue was purified by column chromatography over basic alumina (EtOAc). Yield: 90%; ^1H NMR (300 MHz,

CDCl$_3$, TMS): δ = 7.46-7.43 (d, 2H), 7.31-7.28 (d, 2H), 5.37 (s, 1H), 4.13-3.96 (q, 4H), 3.78-3.62 (m, 4H), 3.18-3.11 (d, 2H), 1.36-1.21 (t, 6H), 0.79 (s, 6H) ppm; MS (FAB): *m/z* calculated for $C_{17}H_{27}O_5P$ is 342.37; found 342.16.

Preparation of 3,4-bis(dodecyloxy)benzaldehyde (7): A mixture of 3,4,-dihydroxybenzaldehyde (0.5 g, 3.62 mmol), K_2CO_3 (5 g) was mixed well in DMF (20 mL). Then 1-bromododecane (2.17 g, 8 mmol) was added drop wise and the reaction mixture was heated at 70 ºC for 10 h with stirring. The reaction mixture was cooled to room temperature and poured into ice-cold water and the precipitate was filtered. The crude precipitate was then passed through a silica gel column using 5% ethyl acetate-*n*-hexane as an eluent. The product was then reprecipitated from CH_2Cl_2 by adding excess methanol. Yield: 95%; ^{1}H NMR (300 MHz, CDCl$_3$, TMS): δ = 9.83 (s, 1H), 7.42-7.40 (m, 2H), 6.96-6.95 (d, 1H), 4.13-4.06 (m, 4H), 1.83-1.75 (m, 4H), 1.48-1.26 (m, 36H), 0.88-0.86 (t, 9H) ppm; MS (FAB): *m/z* calculated for $C_{31}H_{54}O_3$ is 474.76; found 474.31.

Preparation of (*E*)-2-(4-(3,4-bis(dodecyloxy)styryl)phenyl)-5,5-dimethyl-1,3-dioxane (8): Compound **7** (0.4 g, 0.84 mmol) and **5** (0.35 g, 1.02 mmol) were taken in a two neck round bottom flask and kept under argon atmosphere. Dry THF (30 mL) was added to the flask using a pressure equalizer. To this solution NaH was added and stirred overnight at room temperature. After checking the completion of the reaction by TLC, the reaction mixture was concentrated under reduced pressure. The residue thus obtained was extracted with chloroform, washed with water and dried over anhydrous Na_2SO_4. The solvent was removed under reduced pressure. The product was then precipitated from chloroform by adding excess amount of methanol. The crude product thus obtained was further purified by column chromatography over silica gel (40% CHCl$_3$-*n*-hexane). Yield: 85%; ^{1}H NMR (300 MHz, CDCl$_3$, TMS): δ = 7.92-7.91 (m, 2H), 7.60-7.45 (m, 4H), 6.89-6.86 (m, 3H), 5.32 (s, 1H), 3.95-3.86 (m, 4H), 3.70-3.45 (m, 4H), 1.64-1.21 (m, 40H), 0.82-0.81 (t, 9H), 0.79 (s, 6H) ppm; MS (FAB): *m/z* calculated for $C_{56}H_{94}O_5$ is 663.02; found 663.45.

Preparation of (*E*)-4-(3,4-bis(dodecyloxy)styryl)benzaldehyde (9): Compound **8** (0.5 g, 0.59 mmol) and trifluoroacetic acid (TFA) dissolved in CH_2Cl_2 (30 mL) was stirred at room temperature for 5 h. The organic layer was then washed with water dried over anhydrous Na_2SO_4 and evaporated to dryness under reduced pressure. The product was then purified by column chromatography over silica gel (30% CHCl$_3$-hexane). Yield: 90%; ^{1}H NMR (300 MHz, CDCl$_3$, TMS): δ = 9.99 (s, 1H), 7.92-7.91 (m, 2H), 7.7- 7.54 (m, 4H), 6.89-6.86 (m, 3H), 4.05-

3.96 (t, 4H), 1.83-1.75 (m, 4H), 1.48-1.26 (m, 36H), 0.88-0.86 (t, 6H) ppm; MS (FAB): *m/z* calculated for $C_{51}H_{84}O_4$ is 576.89; found 577.50.

Preparation of 1,2-bis(dodecyloxy)-4-((*E*)-4-((*E*)-4-nitrostyryl)styryl)benzene (10): The compound **9** (0.08 g, 0.14 mmol) and diethyl(4-nitrobenzyl)phosphonate (0.082 g, 0.30 mmol) were taken in a two necked round bottom flask and kept under argon atmosphere. The compounds were dissolved in dry THF and NaH (20 mg) was added. The reaction mixture was kept for stirring at room temperature for 2 h. After completion of the reaction, the content was concentrated under reduced pressure. The residue thus obtained was extracted with chloroform, washed with water and dried over anhydrous Na_2SO_4. The solvent was removed under reduced pressure. The product was then precipitated from chloroform by adding excess amount of methanol. The crude product obtained further purified by column chromatography over silica gel (30% $CHCl_3$-hexane). Yield: 80%; ^{1}H NMR (300 MHz, $CDCl_3$, TMS): δ = 8.28-8.21 (m, 2H), 7.71-7.63 (m, 2H), 7.53 (s, 4H), 7.18-7.10 (m, 2H), 7.04-6.95 (m, 2H), 6.95- 6.73 (m, 3H), 4.05-3.98 (t, 4H), 1.83-1.76 (t, 4H), 1.56-1.27 (m, 37H), 0.88-0.86 (t, 6H) ppm; MS (FAB): *m/z* calculated for $C_{58}H_{89}NO_5$ is 696.01; found 697.10.

Preparation of 4-(2-{4-[2-(3,4-bis-dodecyloxyphenyl)-vinyl]-phenyl}-vinyl)-phenylamine (11): The OPV nitro compound (**10**) (0.7 g, 1.01 mmol), $SnCl_2{\cdot}2H_2O$ (0.31 g, 1.36 mmol) in distilled THF containing four drops of 37% HCl was refluxed at 70 °C for 4 h. The reaction mixture was diluted with 50 mL CH_2Cl_2 and washed once with 0.1 M sodium bicarbonate solution and two times with water. The extract collected was dried over anhydrous Na_2SO_4. The organic layer was then filtered through a silica pad using CH_2Cl_2 as the eluent to afford the OPV amino compound (**11**). The product was then collected by precipitation from CH_2Cl_2 using excess methanol. Yield: 85%; ^{1}H NMR (300 MHz, $CDCl_3$, TMS): δ = 7.50-7.28 (m, 4H), 7.19-7.07 (m, 2H), 7.04-6.88 (m, 3H), 6.81-6.79 (m, 3H), 6.40-6.38 (d, 3H), 4.69 (s, 2H), 3.99-3.90 (t, 4H), 1.83-1.76 (t, 4H), 1.51-1.30 (m, 37H), 0.88-0.87 (t, 6H) ppm; MS (FAB): *m/z* calculated for $C_{58}H_{91}NO_3$ is 850.35; found 851.10.

Synthesis of *C*$_3$OPV: To a solution of OPV amine **11** (0.2g, 0.15mmol) and triethylamine (0.5 ml) in dry CH_2Cl_2 (10 mL) a solution of 1,3,5-benzenetricarboxylic acid chloride (0.012g, 0.045 mmol) in dry CH_2Cl_2 (20 mL) was added dropwise. The reaction mixture was stirred for 6 h at room temperature. After completion of the reaction, the solvent was evaporated *in vacuo.* Purification was carried out first by silica gel column chromatography (CH_2Cl_2/hexane 3:1 v/v) followed by size–exclusion chromatography on Bio–Rad BioBeads S–X1 (CH_2Cl_2). The product

was subjected to precipitate from CH_2Cl_2/methanol to give ***C*₃OPV** as a yellow colored solid. Yield: 50%; FT-IR (KBr): ν_{max} = 1260, 1518, 1661, 2854, 2926, 3448 cm^{-1}; ^1H NMR (300 MHz, d_8-THF, TMS): δ = 9.94 (s, 3H), 8.78 (s, 3H), 7.9-7.87 (d. 6H), 7.77-7.41 (m, 16H), 7.19-7.03 (m, 14H), 6.78 (s, 6H), 4.02-3.82 (m, 12H), 1.77-1.31 (m, 180H) 0.89 (s, 18H) ppm; ^{13}C NMR (125 MHz, d_8-THF): δ = 11.59, 20.73, 22.22, 29.60, 29.80, 31.80, 65.20, 73.80, 102.80, 118.34, 124.61, 124.80, 125.40, 1340, 136.70, 138.10, 153.50, 165.20 ppm; MS (MALDI-TOF): *m*/*z* calculated for $C_{183}H_{273}N_3O_{12}$ $[M+H]^+$: 2155.20, found: 2155.23.

1.4.2. Synthesis of *C*₃PBI

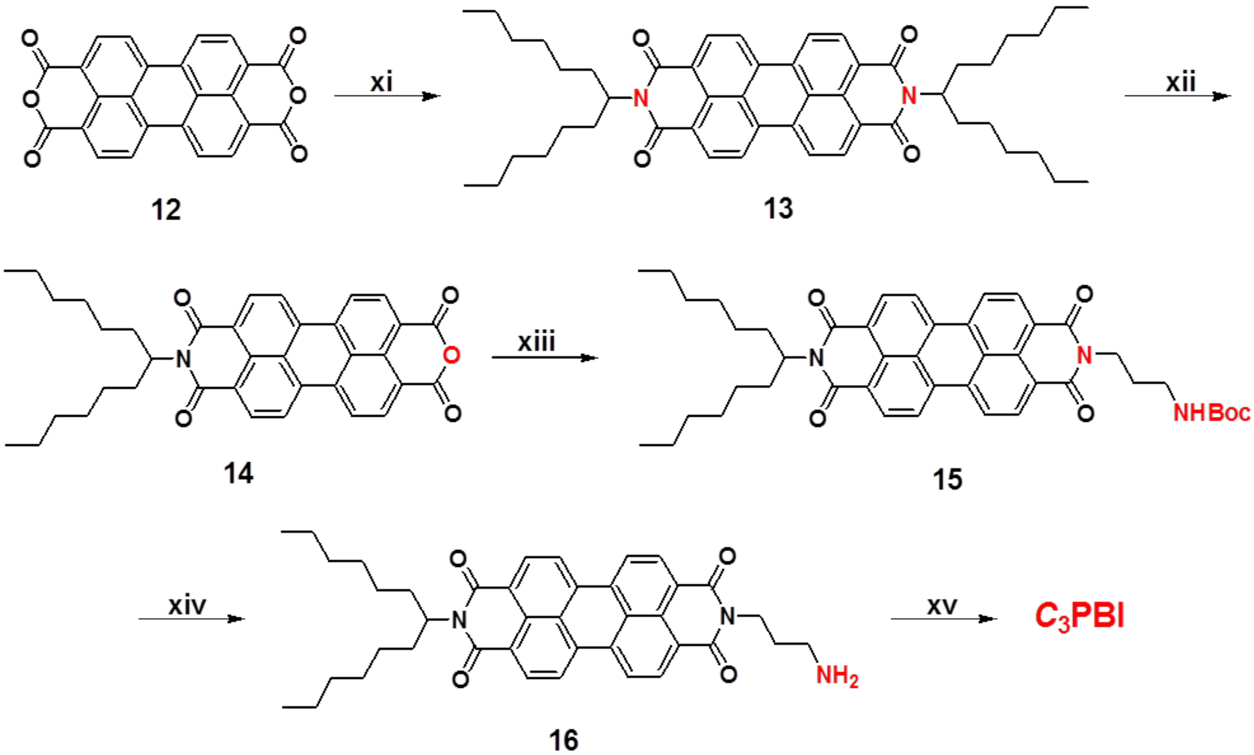

Scheme S2. Reagents and conditions: xi) 1-hexylheptylamine, $Zn(OAc)_2$, imidazole, 160 °C, 2 h, 65%; xii) KOH, *tert*-butanol, 90 °C, 30 min, 40%; xiii) *N*-Boc-1,3-propanediamine, $Zn(OAc)_2$, dry DMF, 95 °C, 18 h, 52%; xiv) TFA, CH_2Cl_2, r.t., 4 h, 77%; xv) 1,3,5-benzenetricarboxylic acid chloride, Et_3N, dry CH_2Cl_2, r.t., 6 h, 50%.

Synthesis of *N*,*N*'-bis(1-hexylheptyl)-3,4,9,10-perylene dicarboxamide (13): A mixture of perylene-3,4,9,10-tetracarboxylic dianhydride (**12**) (0.392 g, 1 mmol), zinc acetate (0.165 g, 0.75 mmol), imidazole (4 g) as solvent and the 1-hexylheptylamine[S1] (0.6 g, 3 mmol) was vigorously stirred at 160 °C for 2 h. After cooling to room temperature, the mixture was dissolved in minimum amount of THF and precipitated in 300 ml 2 N HCl/methanol 2:1 v/v. The precipitate was filtered and washed with water followed by methanol, dried at 80 °C under vacuum. The

crude product was further purified by column chromatography over silica gel (50% $CHCl_3$/hexane). Yield: 65%; ^{1}H NMR (300 MHz, $CDCl_3$, TMS): δ = 8.70-8.64 (m, 8H), 5.21-5.15 (m, 2H), 2.27-2.21 (m, 4H), 1.87-1.83 (m, 4H), 1.33-1.22 (m, 32H), 0.83-0.81 (t, 12H) ppm. MS (FAB): *m/z* calculated for $C_{50}H_{62}N_2O_4$ is 755.04; found 755.23.

Synthesis of N-(1-hexylheptyl)perylene-3,4,9,10-tetracarboxylic-3,4-anhydride-9,10-imide (14): In a 250 mL round bottom flask, **14** (0.5 g, 0.662 mmol) was suspended in 70 mL *tert*-butanol and was treated with of 85% KOH. The reaction mixture was heated with vigorous stirring to reflux until the solution turned dark purple for approximately 30 minutes. The mixture cooled to room temperature, treated with 80 mL acetic acid and 40 mL 2 N HCl and stirred overnight. The dark red precipitate filtered washed with water and dried at 130 °C. The solid was suspended in 150 mL 10% K_2CO_3 solution and refluxed for 30 minutes. The mixture was then cooled to room temperature and filtered. The filter cake was washed with warm 10% K_2CO_3 until the filtrate was clear, rinsed twice with approximately 100 mL 2 N HCl and rinsed thoroughly with water and dried at 130 °C. The solid was then suspended in 100 mL boiling water and triethylamine was added until a dark purple solution of the desired product was formed. Remaining starting material was filtered off and the dark filtrate was acidified with 30 ml 2 N HCl and stirred overnight. The resulting dark precipitate was filtered and rinsed with water and dried. Yield: 40%; ^{1}H NMR (300 MHz, $CDCl_3$, TMS): δ = 8.73-8.67 (m, 6H), 8.01 (m, 2H), 5.21-5.15 (m, 1H), 2.27-2.21 (m, 2H), 1.87-1.83 (m, 2H), 1.33-1.22 (m, 16H), 0.83-0.81 (t, 12H) ppm; MS (FAB): *m/z* calculated for $C_{37}H_{35}NO_5$ is 573.68; found 574.10.

Synthesis of (15): Under argon atmosphere **15** (0.1 g, 0.174 mmol), *N*-Boc-1,3-propanediamine (0.029 g, 0.13 mmol), and zinc acetate (~ 0.01 g) were dissolved in dry DMF (30 mL) and heated at 95 °C for 18 h. After evaporation of the solvent *in vacuo*, the product was dissolved in chloroform and washed with an aqueous citric acid solution followed by brine. The organic layer was dried over anhydrous Na_2SO_4 and evaporated to dryness under reduced pressure. The resulting red solid was subjected to column chromatography over silica gel (0.5-1% methanol in chloroform). Yield: 52%; MS (FAB): *m/z* calculated for $C_{45}H_{51}N_3O_6$ is 729.90; found 732.01.

Synthesis of (16): To a solution of **16** (0.5 g, 0.69 mmol) in CH_2Cl_2, trifluoroacetic acid (10 mL) was added and the mixture was stirred for 4 h at room temperature. After evaporation of the solvent *in vacuo*, the product was dissolved in CH_2Cl_2 and subsequently washed with an aqueous 10% $NaHCO_3$ solution and brine. The organic layer was dried over anhydrous Na_2SO_4

and finally evaporated *in vacuo*. Yield: 77%; ^{1}H NMR (300 MHz, CD_3CN, TMS): δ = 8.46-8.43 (m, 6H), 8.39-8.38 (m, 2H), 5.21-5.15 (m, 1H), 4.22-4.2 (t, 2H), 3.24 (m, 2H), 2.26-2.08 (m, 4H), 1.89 (m, 2H), 1.34-1.26 (m, 16H), 0.85-0.83 (t, 6H) ppm; MS (FAB): *m/z* calculated for $C_{40}H_{43}N_3O_4$ is 629.79; found 630.43.

Synthesis of C_3PBI (18): To a solution of **17** (0.4 g, 0.63 mmol) and triethylamine (0.5 mL) in dry CH_2Cl_2 (10 mL) a solution of 1,3,5-benzenetricarboxylic acid chloride (0.56 g, 0.217 mmol) was added dropwise. The mixture was stirred for 6 h and the solvent was evaporated *in vacuo*. Purification was carried out first by silica gel column chromatography (CH_2Cl_2/hexane 3:1 v/v) followed by size–exclusion chromatography on Bio–Rad BioBeads S–X1 (CH_2Cl_2). The product was subjected to precipitate from CH_2Cl_2/methanol to give ***C_3*PBI** as a red color solid. Yield: 50%; FT-IR (KBr): ν_{max} = 1256, 1347, 1530, 1590, 1658, 2865, 2952, 3079, 3289, 3639 cm^{-1}; ^{1}H NMR (300 MHz, d_8-THF, TMS): δ = 8.72 (s, 3H), 8.44-8.41 (d, 12H), 8.19-8.16 (d, 12H), 7.74 (s, 3H), 5.17 (m, 3H), 4.45-4.35 (t, 6H), 3.74-3.64 (t, 6H), 1.87-1.82 (t, 3H), 1.57-1.37 (m, 62H), 0.85 (s, 18H) ppm; MALDI-TOF-MS: *m*/*z* calculated for $C_{129}H_{129}N_9O_{15}$ $[M+H]^+$: 2046.47, found: 2047.18.

1.5. Isodesmic or Equal-K Self-Association Model

The temperature dependent absorption spectrum of ***C_3*OPV** is fitted with isodesmic or equal-K model in which the binding constant for each addition of monomer to the growing assembly is the same. Standard isodesmic model is used for analyzing the data.[S2] According to this model, the degree of polymerization or the molar fraction of aggregated species $\alpha_{agg}(T)$ is given by the equation (1).

$$\alpha_{agg}(T) \cong \frac{1}{1 + \exp\left(-0.908\Delta H \frac{T-T_m}{RT_m{}^2}\right)} \qquad (1)$$

By using equation (1), T_m, the melting temperature defined as the temperature for which α_{agg} = 0.5 and ΔH, the molar enthalpy release related to the formation of noncovalent intermolecular interactions were determined. The number-averaged degree of polymerization DP_N can be calculated from $\alpha_{agg}(T)$:

$$DP_N = \frac{1}{\sqrt{1-\alpha_{agg}(T)}} \qquad (2)$$

This expression can be related to the equilibrium constant *K* and the total concentration of molecules C_T via:

$$DP_N = \frac{1}{\sqrt{1-\alpha_{agg}(T)}} = \frac{1}{2} + \frac{1}{2}\sqrt{4K_e(T)C_T + 1} \quad (3)$$

1.6. Nucleation-Elongation Model

In order to demonstrate the involvement of nucleation and growth process in the observed self-assembly of ***C*₃PBI**, we have attempted to analyze the curve on the basis of the model proposed by van der Schoot, Schenning and Meijer.[S3] According to this model, in the *elongation* regime, the fraction of aggregated species (α_{agg}) can be defined by the following equation:

$$\alpha_{\mathrm{agg}} = \alpha_{\mathrm{SAT}}\left(1 - \exp\left[\frac{-\Delta H_e}{RT_e^2}(T - T_e)\right]\right) \quad (4)$$

where, ΔH_e is the enthalpy corresponding to the aggregation (elongation) process, T the absolute temperature, T_e the elongation temperature, R the ideal gas constant. α_{SAT} is a parameter introduced to ensure that $\alpha_{agg}/\alpha_{SAT}$ does not exceed unity.

On the other hand in the *nucleation* regime the fraction of aggregated species (α_{agg}) can be defined by:

$$\alpha_{\mathrm{agg}} = K_a^{1/3} \exp\left[\left(2/3\, K_a^{-1/3} - 1\right)\frac{h_e}{RT_e^2}(T - T_e)\right] \quad (5)$$

where K_a is the dimensionless equilibrium constant of the activation step at the elongation temperature.

The average length of the stack $\langle N_n \rangle$ averaged over the nucleated species at the T_e is given by:

$$\langle N_n(T_e)\rangle = \frac{1}{(K_a)^{1/3}} \quad (6)$$

The substitution of K_a in equation 6 enables the calculation of the number of aggregated molecules, *i.e.*, the nucleus size, at the elongation temperature.

2. Additional Figures

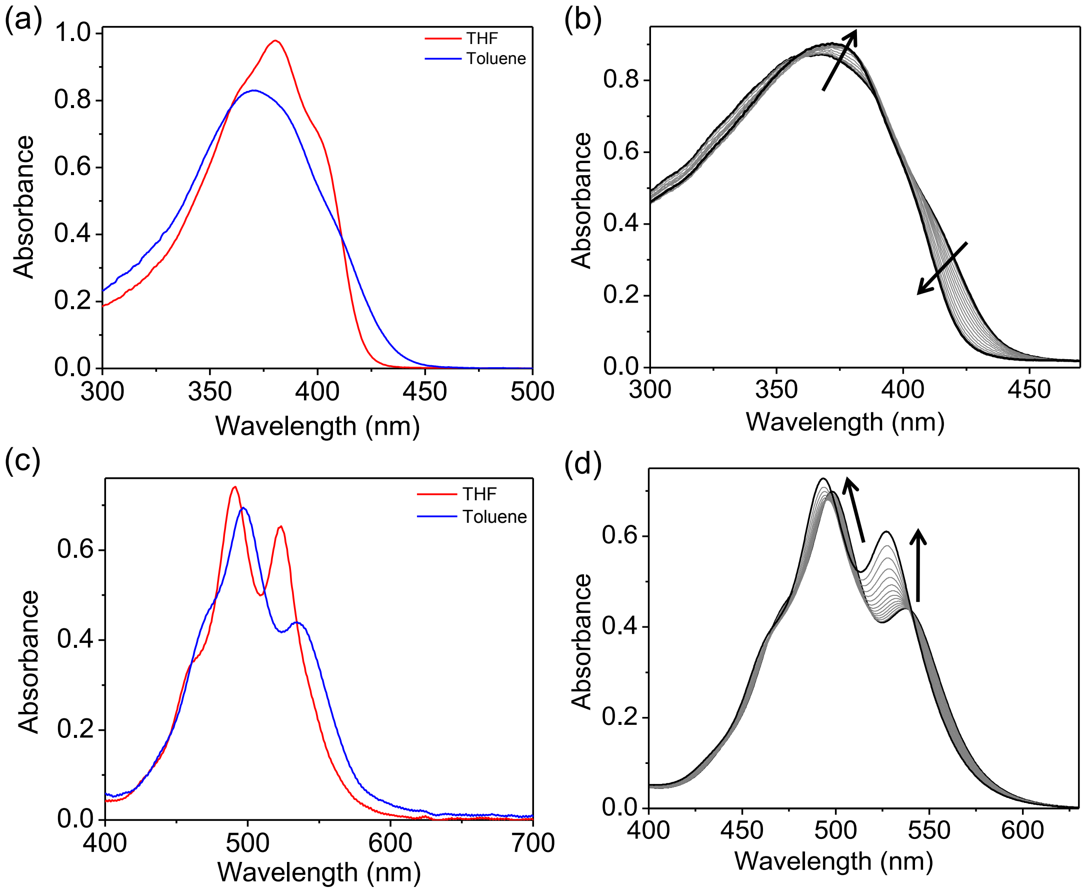

Fig. S1 UV/Vis absorption spectra of (a) ***C*₃OPV** and (c) ***C*₃PBI** in THF and toluene (1 × 10^{-4} M). Temperature dependent absorption spectra of (b) ***C*₃OPV** and (d) ***C*₃PBI**, in toluene (1 × 10^{-4} M). Arrows indicates relative changes in absorption with increase in temperature from 20 to 90 °C.

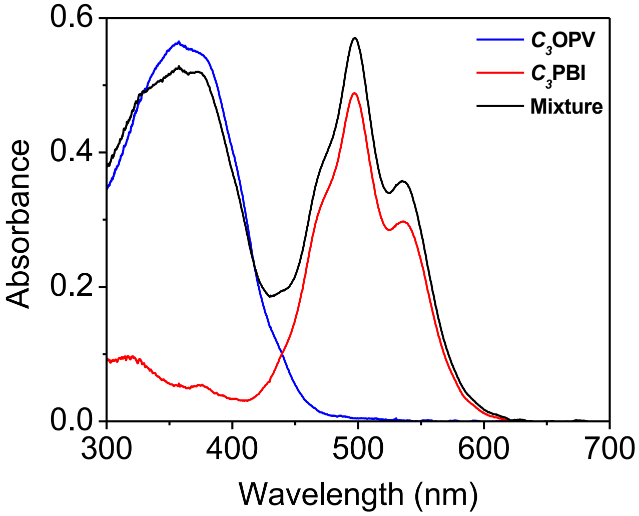

Fig. S2 Comparison of the absorption spectra of ***C*3OPV**, ***C*3PBI** and 1:1 mixture in toluene. The concentration of both components is 1 × 10^{-4} M.

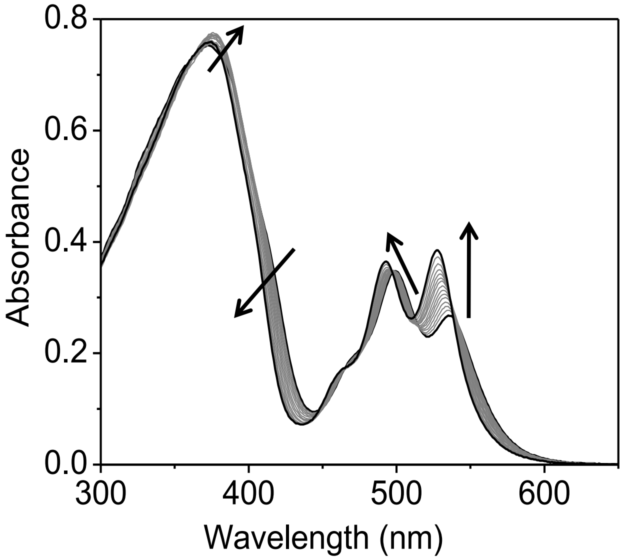

Fig. S3 Temperature dependent UV/Vis absorption spectra of 1:1 mixture of ***C*3OPV** and ***C*3PBI** in toluene (1 × 10^{-4} M). Arrows indicates relative changes in absorption with increase in temperature from 20 to 90 °C.

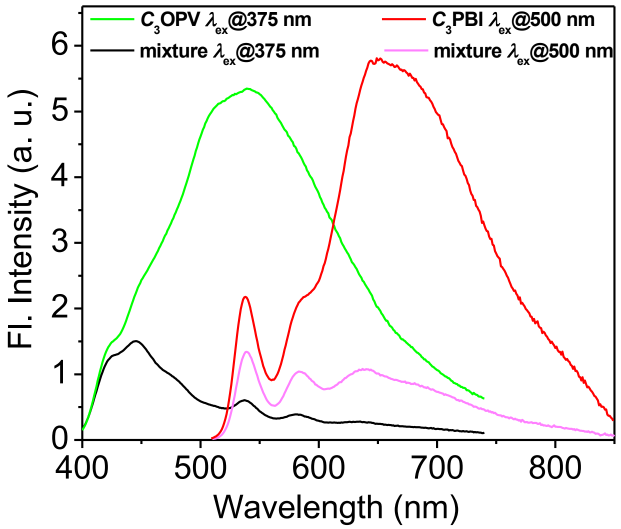

Fig. S4 Emission spectra of ***C*₃OPV**, ***C*₃PBI** and 1:1 mixture in toluene (1×10^{-4} M).

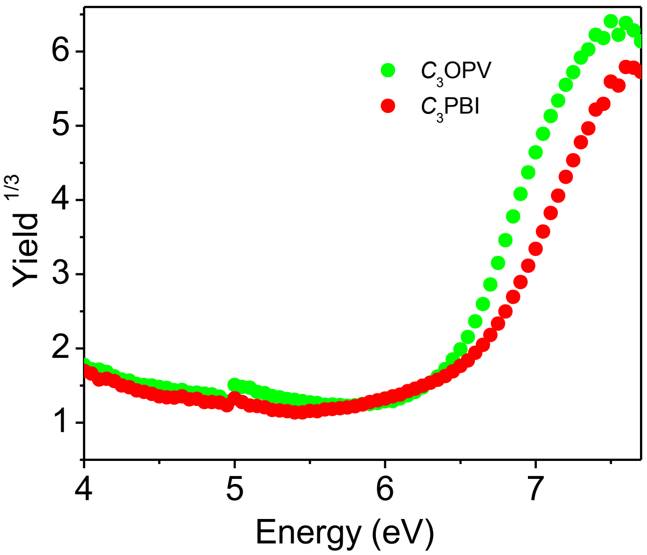

Fig. S5 Photoelectron yield spectra in air for ***C*₃OPV** and ***C*₃PBI** films over an ITO coated glass plates drop cast from toluene solutions.

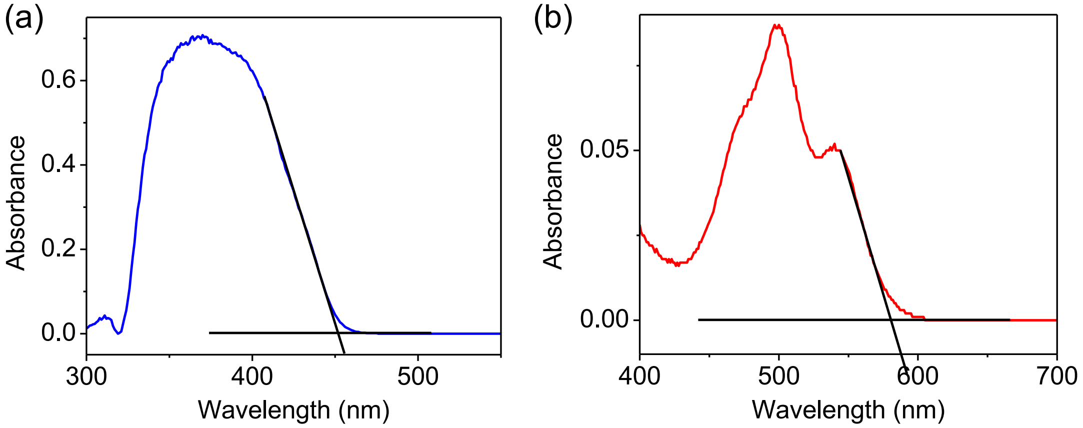

Fig. S6 Absorption spectra of (a) ***C*₃OPV** and (b) ***C*₃PBI** in the film state. The tangents are drawn in order to get the onset value (λ_{onset} for ***C*$_3$OPV** = 451.62 nm, λ_{onset} for ***C*$_3$PBI** = 580.75 nm).

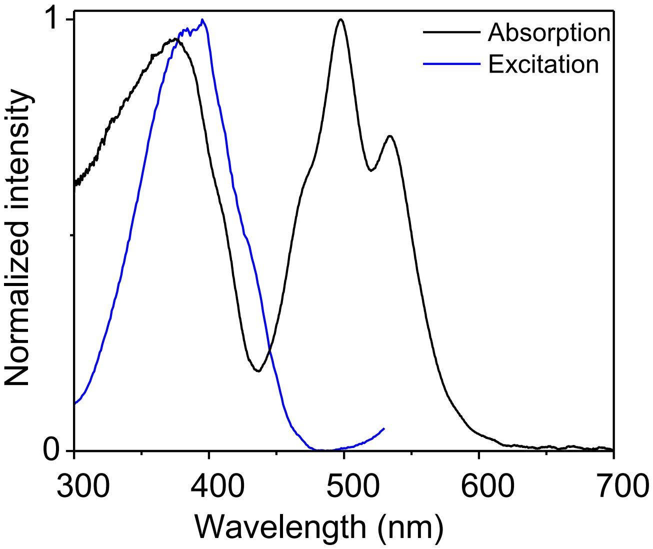

Fig. S7 Comparison of the absorption and the excitation spectra of 1:1 mixture of ***C*$_3$OPV** and ***C*$_3$PBI** in toluene (emission monitored at 540 nm).

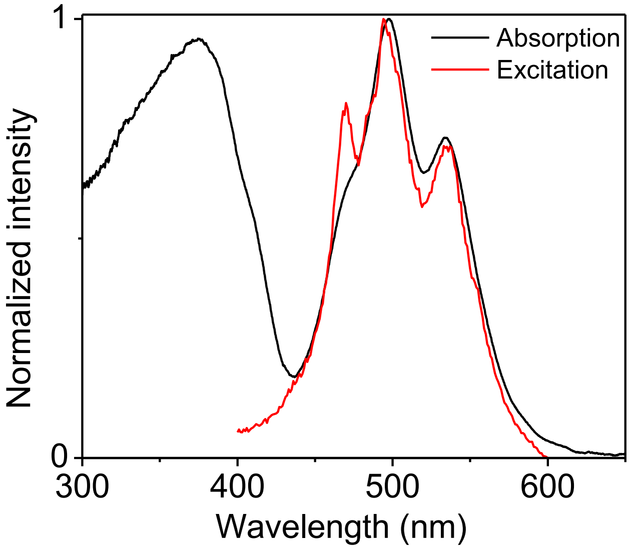

Fig. S8 Comparison of the absorption and the excitation spectra of 1:1 mixture of ***C*₃OPV** and ***C*₃PBI** in toluene (emission monitored at 650 nm).

3. Supporting Information References

S1. J. Tadanier, R. Hallas, J. R. Martin and R. S. Stanaszek, *Tetrahedron*, 1981, **37**, 1309-1316.

S2. (*a*) R. B. Martin, *Chem. Rev*. 1996, **96**, 3043–3064; (*b*) Z. Chen, A. Lohr, C. R. Saha-Möller, F. Würthner, *Chem. Soc. Rev.*, 2009, **38**, 564–584.

S3. (*a*) M. M. J. Smulders, A. P. H. J. Schenning and E. W. Meijer, *J. Am. Chem. Soc.*, 2008, **130**, 606–611; (*b*) P. Jonkheijm, P. van der Schoot, A. P. H. J. Schenning, E. W. Meijer, *Science*, 2006, **313**, 80–83.
